# Bleaching Susceptibility and Recovery of Colombian Caribbean Corals in Response to Water Current Exposure and Seasonal Upwelling

**DOI:** 10.1371/journal.pone.0080536

**Published:** 2013-11-25

**Authors:** Elisa Bayraktarov, Valeria Pizarro, Corvin Eidens, Thomas Wilke, Christian Wild

**Affiliations:** 1 Coral Reef Ecology Group (CORE), Leibniz Center for Tropical Marine Ecology (ZMT), Bremen, Germany; 2 Center of Excellence in Marine Sciences (CEMarin), Santa Marta, Magdalena, Colombia; 3 Department of Animal Ecology & Systematics, Justus Liebig University Giessen, Giessen, Hessen, Germany; Pennsylvania State University, United States of America

## Abstract

Coral bleaching events are globally occurring more frequently and with higher intensity, mainly caused by increases in seawater temperature. In Tayrona National Natural Park (TNNP) in the Colombian Caribbean, local coral communities are subjected to seasonal wind-triggered upwelling events coinciding with stronger water currents depending on location. This natural phenomenon offers the unique opportunity to study potential water current-induced mitigation mechanisms of coral bleaching in an upwelling influenced region. Therefore, coral bleaching susceptibility and recovery patterns were compared during a moderate and a mild bleaching event in December 2010 and 2011, and at the end of the subsequent upwelling periods at a water current-exposed and -sheltered site of an exemplary bay using permanent transect and labeling tools. This was accompanied by parallel monitoring of key environmental variables. Findings revealed that in 2010 overall coral bleaching before upwelling was significantly higher at the sheltered (34%) compared to the exposed site (8%). Whereas 97% of all previously bleached corals at the water current-exposed site had recovered from bleaching by April 2011, only 77% recovered at the sheltered site, but 12% had died there. In December 2011, only mild bleaching (<10% at both sites) was observed, but corals recovered significantly at both sites in the course of upwelling. No differences in water temperatures between sites occurred, but water current exposure and turbidity were significantly higher at the exposed site, suggesting that these variables may be responsible for the observed site-specific mitigation of coral bleaching. This indicates the existence of local resilience patterns against coral bleaching in Caribbean reefs.

## Introduction

Climate change can impede scleractinian corals in their role as reef ecosystem engineers [1], [2] mainly because mass coral bleaching, one of the main consequences of climate change-induced ocean warming, negatively impacts growth and health of affected corals [3].

In 2005, such a mass coral bleaching event affected 80% of all Caribbean reef corals, after which 40% died, when thermal stress exceeded any recorded data on seawater temperature of the last 20 years [4]. This particular mass coral bleaching event was later termed the “Caribbean Crisis” [5]. However, local differences were considerable. In the Colombian Caribbean, up to 80% of all hard corals showed signs of bleaching in locations such as Islas del Rosario (Cartagena) or Islas San Bernardo [6]. In contrast, no severe bleaching occurred for coral communities in the bays of the Tayrona National Natural Park (TNNP) near the city of Santa Marta before 2010 [6], [7]. During the “Caribbean Crisis” in 2005, only 1 – 5% of the coral cover in TNNP was affected by bleaching with a negligible mortality of below 1% [6].

It remains uncertain why hard corals in the TNNP were not affected by the “Caribbean Crisis”. This area is of high interest and suitable for studies on adaptation potential of corals as it is influenced by pronounced changes of environmental variables between a rainy season with high precipitation and riverine discharge and a dry season that goes along with seasonal coastal upwelling [8]–[10]. The major rainy season (May-November) is represented by low winds, more than 80% of the annual rainfall, low salinity and increased seawater temperature [8], [11]. Highest seawater temperatures usually occur between October and November and can reach up to 30°C [10].

The TNNP constitutes one of three major upwelling nuclei of the Guajira Upwelling System [11] extending from the Guajira Peninsula, near the border with Venezuela, to the city of Santa Marta in the Colombian Caribbean [9]. The Guajira Upwelling is part of the Southern Caribbean Upwelling System [12]. Upwelling coincides with the major dry season (December-April), during which the bays of TNNP are exposed to strong winds from the Caribbean low-level jet of north-east trade winds [9]. Increased winds parallel to the coast displace humidity and trigger an Ekman transport off the coast and an upwelling of sub-surface waters in the coastal zone [9], [13]. This seasonal upwelling thereby leads to changes in physicochemical variables such as temperature decrease (from 28°C to 21°C) [8], [10], [11] and salinity increase (from 33 to 38) [8], [10]. Increased concentrations of inorganic nutrients and chlorophyll *a* characterize the usually oligotrophic region during non-upwelling as mesotrophic in periods of upwelling [14]. The wind pattern triggering upwelling may lead to a different exposition of higher wave- and water current-impact at all western sides of the TNNP bays as compared to the sheltered eastern sides due to their topographical orientation.

It was first hypothesized by Glynn [15] and later supported by several other studies [16]–[18] that upwelling centers can serve as refuge areas for corals by counteracting seawater temperature increases that may provoke coral bleaching. Further studies provide evidence for reduced coral bleaching in other upwelling-affected regions such as on the western coast of Mexico [19]–[21], the Gulf of Panama [22], [23], the Gulf of Papagayo/Costa Rica [24], the Bahamas [17], South Africa [25], Northern Madagascar [26] and TNNP [6]. Chollett et al. [18] suggested the TNNP region as potential refuge area against coral bleaching as seasonal upwelling events may coincide with severe warming events and consequently offset bleaching impact due to upwelling-induced seawater temperature decrease.

However, so far no studies have addressed the effects of water-current exposition on coral communities in a region influenced by seasonal upwelling. We hypothesize that local differences of coral bleaching mitigation through water current exposure are present in TNNP which make corals at water current-exposed sites less susceptible to bleaching than their water current-sheltered counterparts.

The goal of the present study was therefore to observe coral bleaching susceptibility and recovery patterns between water current-exposed and -sheltered sites of one exemplary bay in TNNP by a detailed monitoring, to compare bleaching incidence over a study period of two years (2010–2012) and to understand possible mechanisms of water current-mediated coral bleaching mitigation in a region with seasonal upwelling. For this purpose, coral bleaching monitoring campaigns (line-transect methods and labeling tools) and measurements of key environmental variables (water temperature, salinity, water currents, inorganic nutrient concentrations, chlorophyll *a*, and water clarity as indirect measure for turbidity) were combined.

## Materials and Methods

### Ethics Statement

All necessary permits were obtained for the described study by Instituto de Investigaciones Marinas y Costeras (INVEMAR) in Santa Marta, Colombia which complied with all relevant regulations.

### Description of Study Site

**Figure 1:**
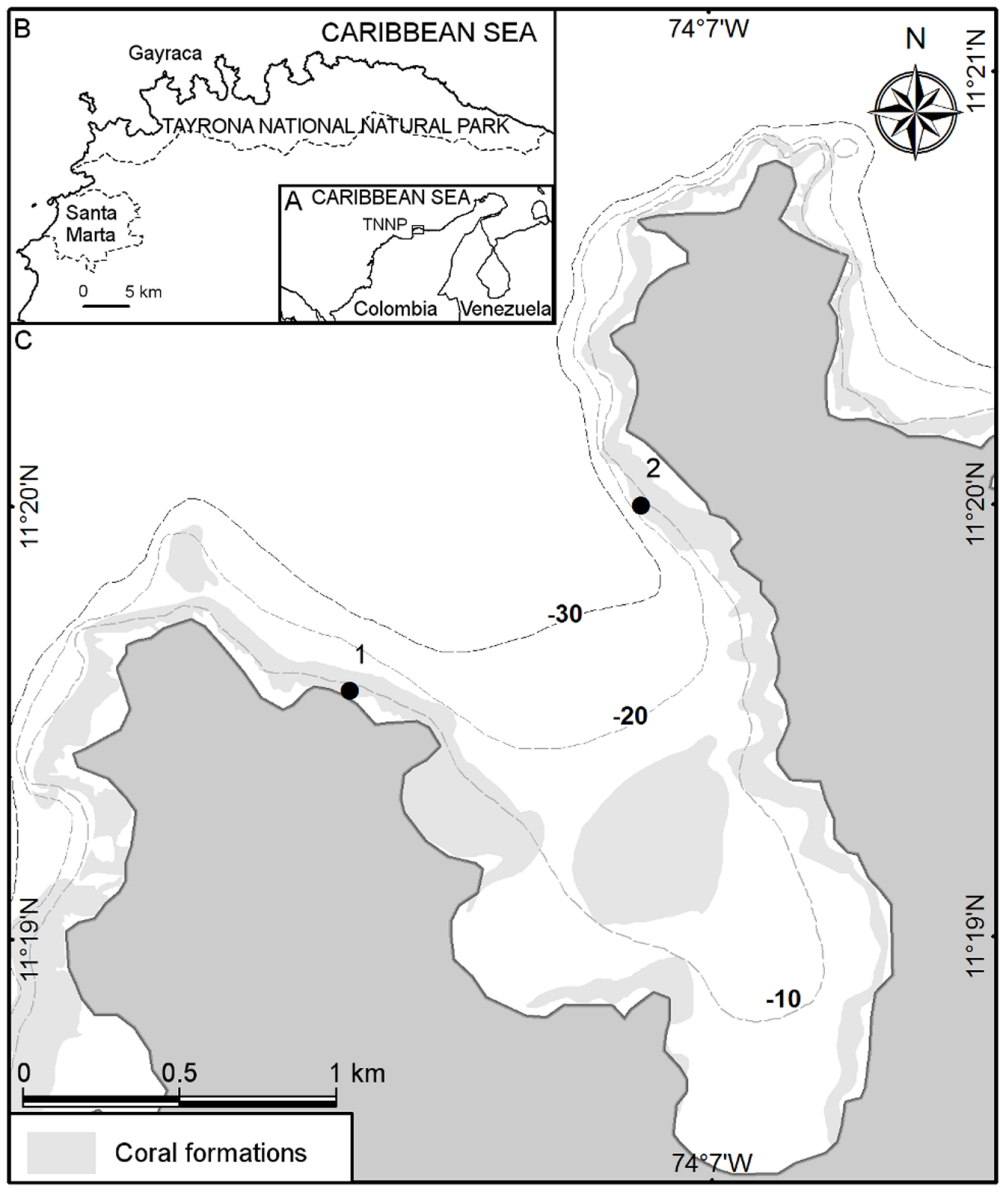
Location of Gayraca Bay and Tayrona National Natural Park (TNNP) in the Caribbean Sea. (A) Location of TNNP. (B) The bays of TNNP and city of Santa Marta. (C) Sampling locations in Gayraca Bay. Circles indicate (1) water current-exposed and (2) -sheltered site. Depth contours are depicted with dashed lines. Source of map: [80].

The TNNP is located at the northern coast of Colombia (Fig. 1A) and represents a biodiversity hotspot in the Colombian Caribbean [27]. The region is affected by a distinct seasonality including a rainy and a dry season during which coastal upwelling occurs. In this study, the months December, January, February, March and April were categorized as upwelling (dry season) and the months May, June, July, August, September, October and November, as non-upwelling (rainy season) according to [8], [11], [28].

The study was carried out in Gayraca Bay, located at 11.33°N, 74.11°W (Fig. 1B). Here, moderate coral bleaching was observed at the beginning of this study in November 2010. Therefore, monitoring of coral communities was initiated in December 2010 and proceeded until April 2012. A water current-exposed site on the western side of the bay with strong impact of winds, waves and water currents, and a water current-sheltered site on the eastern side of the bay were selected for investigation. The sites had a hard coral cover of 41±9% at the exposed and 24±1% at the sheltered site (determined in December 2011), and were located ca. 1 km away from each other (Fig. 1C).

### Coral Bleaching Monitoring

Scleractinian corals were monitored by SCUBA along permanent line-transects at the water current-exposed and -sheltered site (Fig. 1C) in a water depth of 10±1 m. Surveys on coral bleaching were performed in two campaigns and at both sites. Therefore, one campaign consisted in monitoring of coral bleaching before onset of upwelling in December and a repeated monitoring during coral recovery phase end of upwelling in March/April the following year. During the first campaign, monitoring was performed on 5 transects of 10 m length and during the second on 3 transects of 50 m length. Transect replicates were arranged at 1 – 3 m from each other. Coral bleaching was observed after seawater temperature increased (associated with El Niño in 2010) and unusually strong rainfalls (NOAA's Climate Prediction Center, www.cpc.ncep.noaa.gov). Permanent transects were labeled using buoys at beginning and end of each transect as well as by equally spaced marks (every ∼3 m) fixed on the ground throughout each transect. A measuring tape was attached to the buoys and marks during monitoring. Corals were identified at the species level. All surveyed corals were marked by labeling tools (nails with plastic marks indicating colony numbers) during the first campaign and by underwater maps containing exact coral position and species identification during the second campaign in order to survey same coral colonies over time. Coral bleaching and recovery were monitored for each coral colony along the line transects according to BLAGRRA (http://www.agrra.org/BLAGRRA). Coral condition was categorized and scored as normal (0), pale (1), bleached (2) and recently dead/overgrown (3). A lower score denominated a better condition during coral bleaching event or more efficient regeneration during recovery phase.

Site-specific bleaching and mortality indexes (BMI) were calculated according to [29] for each site and coral bleaching monitoring campaign according to the formula:
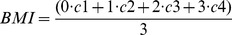
in which the % coral cover of each coral condition category (c_1_ = normal, c_2_ = pale, c_3_ = bleached and c_4_ = recently dead/overgrown) was weighted by their score 0 – 3.

The community bleaching susceptibility index (CBSI) was calculated in analogy to BMI [29], [30] as an index for bleaching response of coral communities at each site taking their specific susceptibility to bleaching into account. Calculations involved 5 species for the exposed and 8 for the sheltered site. Mean benthic cover data of each of these species along 3 transects of 50 m from the second coral bleaching monitoring campaign was used due a more comprehensive representation of the coral community at both sites. The surveyed coral species were ranked according to their reported bleaching susceptibility [31]–[33] into 4 bleaching susceptibility groups (0 – 3). Therefore, *Montastraea cavernosa* (s_1_) accounted for less susceptible corals with no weight (0); *Colpophyllia natans* (s_2_), *Diploria strigosa* (s_3_) and *Diploria labyrinthiformis* (s_4_) were addressed as moderate susceptible with a score of (1); *Porites astreoides* (s_5_) and *Siderastrea siderea* (s_6_) were susceptible with (2), and *Orbicella faveolata* (s_7_) and *Orbicella franksi* (s_8_) (formerly referred to as *Montastraea faveolata* and *Montastraea franksi*, [34]) belonged to corals characterized as highly susceptible with (3) [31], [33]. The following formula for CBSI was modified from Wall et al. [30] where s_1_ to s_8_ represent the live coral cover of each species in %:
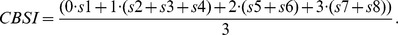



### Monitoring of Environmental Variables

All measurements (water currents) and water samplings (for salinity, inorganic nutrients and chlorophyll *a* concentration) were performed monthly at a water depth of 10 m and in direct vicinity (within a radius of 20 m) to the first coral monitoring transects at both study sites (Fig. 1C). Water temperature was measured continuously *in situ* using calibrated HOBO TidBit v2 temperature loggers (Onset Computer Corp., Bourne, USA) with temporal intervals of 5 min and accuracy of ±0.2°C between October 2010 and May 2012. Therefore, loggers were attached to the reef structure and 10 m water depth at both study sites.

Water current exposure was determined by the clod card technique [35], [36] using gypsum clods (type “stone”, Class III, Dentales America Ldta, Bogotá, Colombia) attached to acrylic plates by water-proof contact cement (Líder Epoxi SinteSolda, Sinteco S. A., Bogotá, Colombia) between February 2012 and February 2013. Time of deployment on the reef structure was 48±2 h (n = 4 per site and month). Still water controls were obtained by inserting clod cards into a closed 35 L bucket with predrilled holes avoiding a saturation of gypsum (n = 4 per month, sheltered site), as described elsewhere [36]. Diffusion factor index (DF) was used as an indirect measure of water current velocity and was obtained by dividing the weight loss of water current exposed gypsum clods by their calm water controls [35]–[37].

Water samples for determination of salinity, inorganic nutrient concentrations (nitrate, nitrite and soluble reactive phosphorus (SRP) mainly present in the form of orthophosphate) and chlorophyll *a* were collected using 3.8 L plastic containers (n = 3 per site and month) from the water column 1 m above the bottom and between January 2011 and February 2013 and 8 – 11 am. Salinity was measured with a portable meter (HQ40d, Hach, Loveland, USA) equipped with a 4-pole conductivity probe (CDC401, Hach, Loveland, USA) with accuracy of ±0.1. Samples were transported on ice and dark to the laboratory, filtered immediately (glass fiber filters, 0.7 µm particle retention, VWR International) and frozen at −20°C until analysis. Inorganic nutrient concentrations from seawater samples were measured spectrophotometrically according to [38]. Chlorophyll *a* from filters was extracted by 6 mL 90% acetone per sample for 24 h at 4°C and dark. Measurements were performed by a fluorometer (10AU™ Field Fluorometer, Turner Designs, Sunnyvale, USA) with a detection limit of 0.025 µg L^−1^.

Measurements of water clarity as an indirect measure of turbidity were performed monthly by Secchi disc with a replication of n = 4 at each site in Gayraca Bay between May 2011 and February 2013. Measurements were conducted at the shady side of the boat at 8 – 11 am.

### Data Analyses

Permutation multivariate analysis of variance (PERMANOVA [39]) was applied for multivariate data of the coral bleaching monitoring and univariate data on monthly monitored environmental variables. Tests were performed using type III sums of squares and 999 permutations under a reduced model. For analysis of coral bleaching, relative proportions of corals characterized as normal, pale, bleached and recently dead/overgrown per transect (variables) were evaluated while each transect represented an independent sample. Site (2 levels: exposed vs. sheltered) and monitoring time (2 levels: before upwelling vs. end of upwelling) were set as fixed factors for analysis. Euclidean similarity distance was applied to create the resemblance matrix for all analyses. For analyses of environmental monitoring variables, univariate PERMANOVA routines were applied after resemblance of monthly means for each variable (temperature, degree-heating days, salinity, water current velocity, nitrate, nitrite, SRP, chlorophyll *a*, and water clarity) and the fixed factors site (2 levels: exposed vs. sheltered) and season (2 levels: upwelling vs. non-upwelling).

Temperature indices (mean, maximum and minimal water temperature) were calculated from *in situ* continuous temperature measurements (every 5 min) and are represented as monthly and seasonal means for both sites during the study period. Degree-heating days were calculated from daily mean temperature data according to [23], in analogy to degree-heating weeks [40], [41]. Seawater temperature above the locally-calculated coral bleaching threshold of 29.4°C [42] was considered as thermal stress.

Statistical analyses were conducted by the software PRIMER© (Plymouth Routines in Multivariate Ecological Research; v 6.1.11 PRIMER-E Ltd., UK) and the PRIMER© add on PERMANOVA+ (v 1.0.1 PRIMER-ELtd., UK). The software SigmaPlot12.0 (Systat Software, Inc) was used for graphical representation of data.

## Results

### Coral Bleaching Monitoring

**Figure 2:**
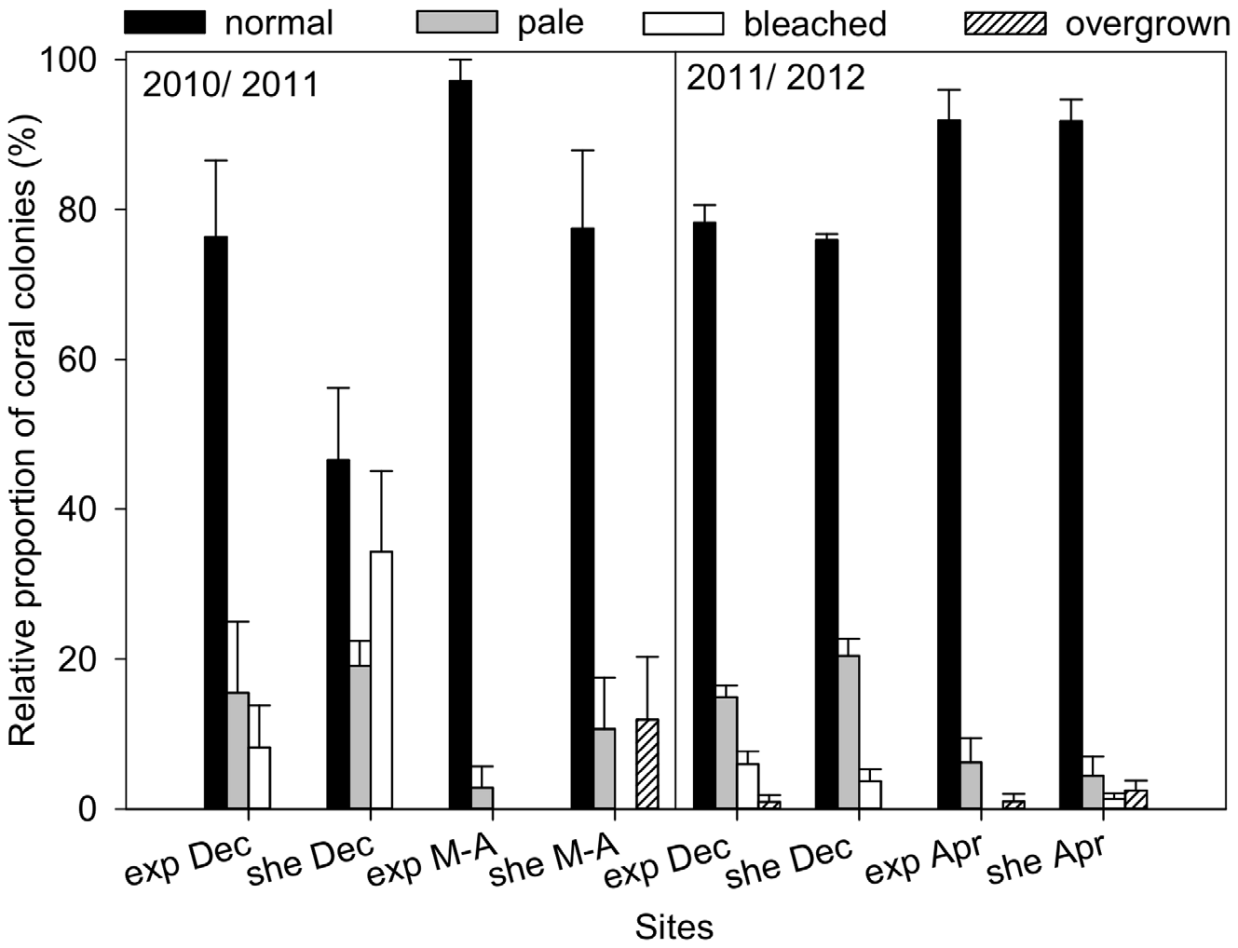
Relative proportion of normal, pale, bleached and overgrown coral colonies. Coral conditions during two coral bleaching monitoring campaigns (December (Dec) 2010-March/April (M-A) 2011 and December 2011-April (Apr) 2012) for the water current-exposed (exp) and -sheltered (she) site in Gayraca Bay along line transects and a water depth of 10 m are illustrated. Replication during first monitoring campaign (2010/2011) accounted for 5 transects of 10 m length and during second monitoring campaign (2011/2012) for 3 transects of 50 m length, respectively. Mean values of transect replicates + SE are displayed.

**Figure 3:**
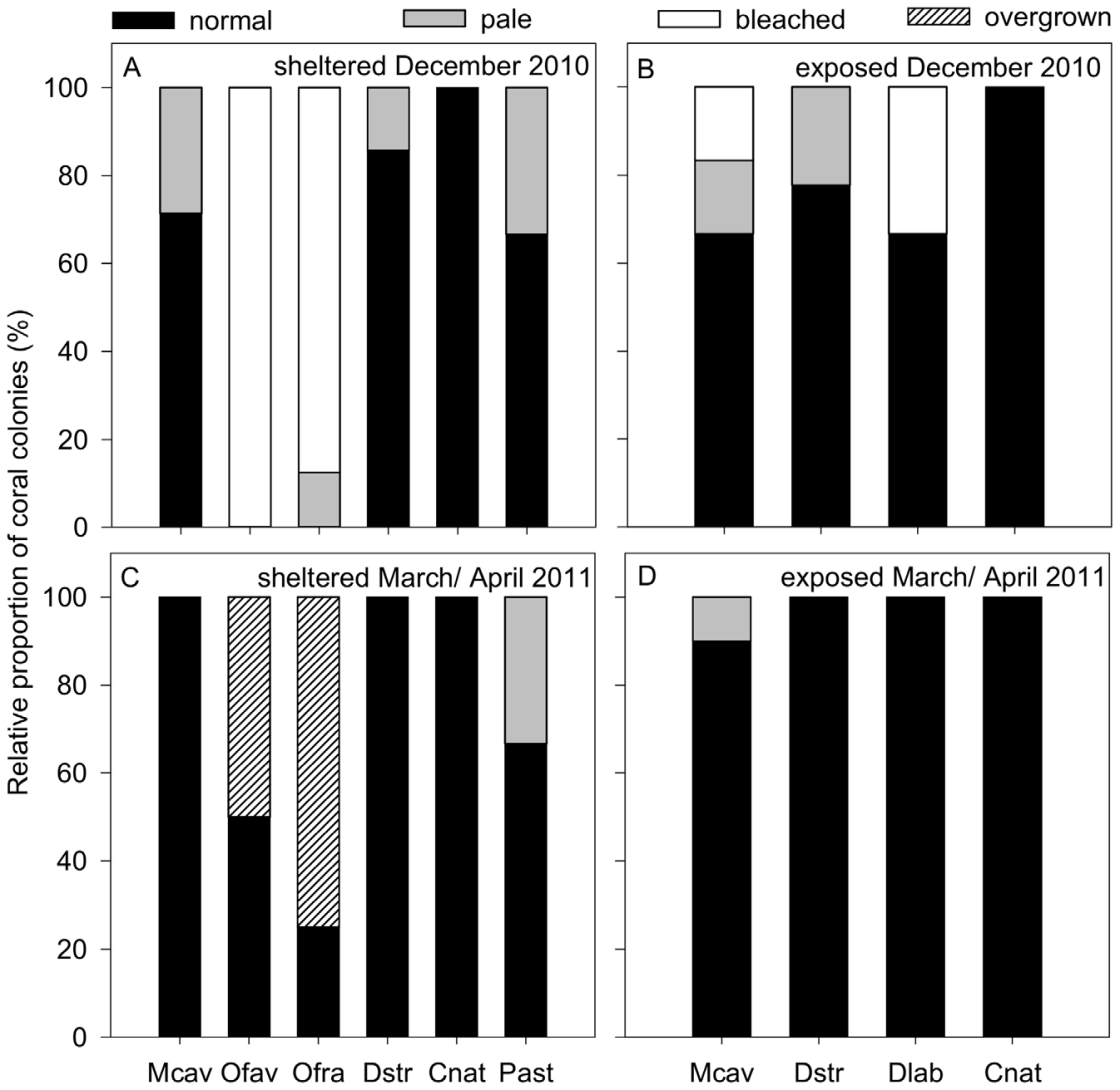
Relative proportion of coral species conditions during the first coral bleaching monitoring campaign. Condition of coral colonies (normal, pale, bleached, overgrown) along line-transects at the water current-exposed and -sheltered site in December 2010 and March/April 2011 in Gayraca Bay and a water depth of 10 m. Coral condition at the sheltered (A) and the exposed (B) site in December 2010 are displayed in the upper panels and (C) and (D) represent the sheltered and exposed site in March/April 2011. Abbreviations: *Montastraea cavernosa* (Mcav), *Orbicella faveolata* (Ofav), *Orbicella franksi* (Ofra), *Diploria strigosa* (Dstr), *Diploria labyrinthiformis* (Dlab), *Colpophylla natans* (Cnat), *Porites astreoides* (Past).

Coral bleaching occurred at the end of 2010 and 2011 in the TNNP region. The bleaching extent was higher in December 2010 (8 – 34%) than in December 2011 (4 – 6%; Fig. 2). During the first coral bleaching monitoring campaign, significant differences were found between sites (Table 1) indicating that the proportion of overall bleached corals was significantly smaller at the exposed compared to the sheltered site (8% vs. 34%, respectively; Fig. 2). Sites had a different community composition with *O. faveolata* and *O. franksi* only occurring at the sheltered site (Fig. 3). Here, in December 2010, 100% of all *O. faveolata* and 88% of *O. franksi* were bleached (Fig. 3A). The content of 19% pale corals were distributed over 29% of all *M. cavernosa*, 13% of *O. franksi*, 14% of *D. strigosa* and 33% of *P. astreoides* (Fig. 3A). At the exposed site, 17% of all *M. cavernosa* and 33% of all *D. labyrinthiformis* were bleached (Fig. 3B). These corals were not observed to bleach at the sheltered site. The remaining 16% pale corals at the exposed site were distributed over 16% of all *M. cavernosa* and 22% *D. strigosa* (Fig. 3B).

**Table 1:** PERMANOVA results for two campaigns of coral bleaching monitoring.

	First coral bleaching monitoring (2010/2011)					Second coral bleaching monitoring (2011/2012)				
	*df*	*SS*	*MS*	Pseudo-*F*	*p*	*df*	*SS*	*MS*	Pseudo-*F*	*p*
Site	1	0.424	0.424	4.917	0.013*	1	0.002	0.002	0.389	0.663
Time	1	0.633	0.633	7.339	0.004**	1	0.120	0.120	24.700	0.003**
Site * time	1	0.117	0.117	1.360	0.271	1	0.006	0.006	1.172	0.296
Residuals	16	1.381	0.086			8	0.039	0.005		
Total	19	2.555				11	0.167			

Levels of significance are indicated by asterisks with * for significant (p<0.05) and ** for very significant (p<0.01). Abbreviations: degrees of freedom (df), sum of squares (SS, type III), mean sum of squares (MS).

**Figure 4:**
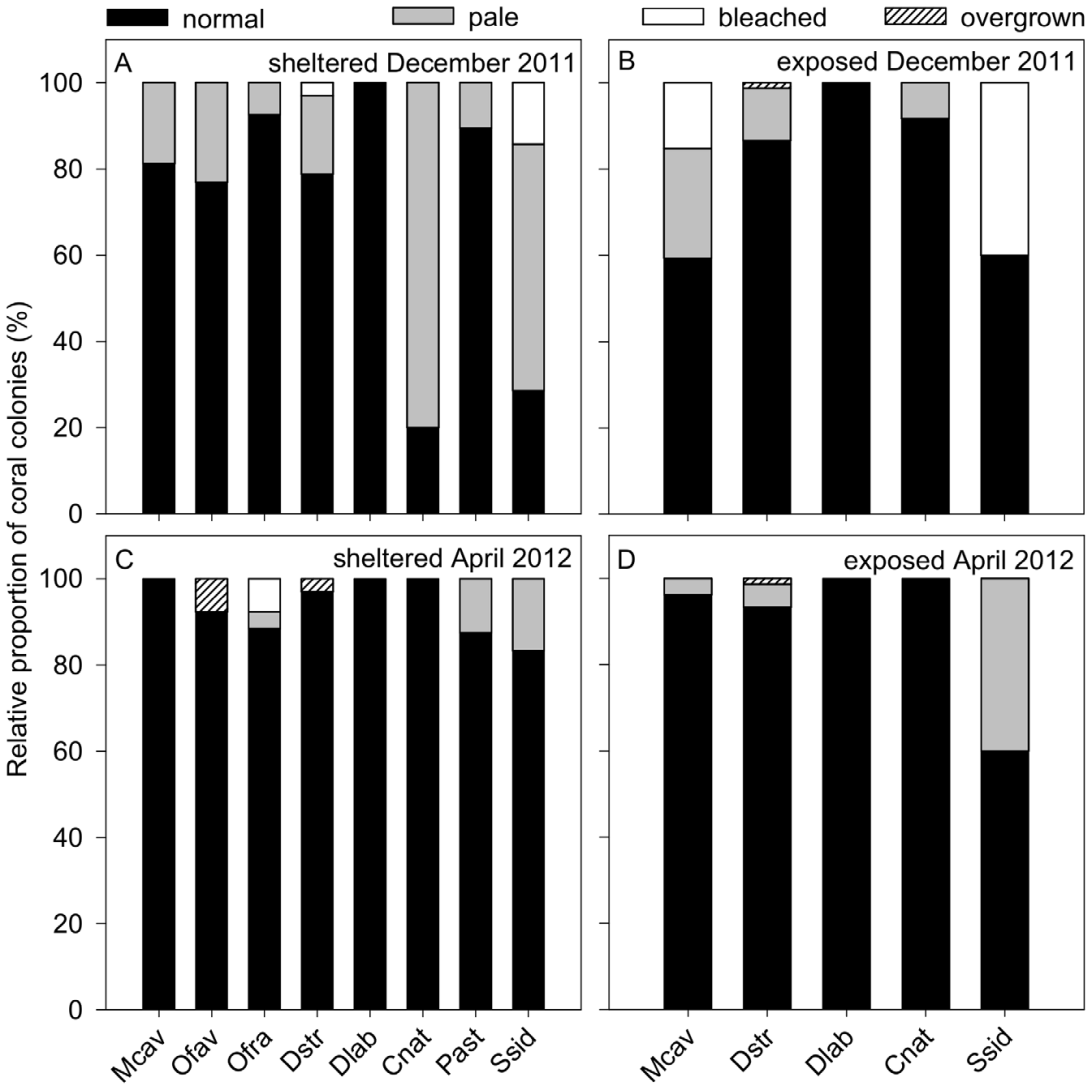
Relative proportion of coral species conditions during the second coral bleaching monitoring campaign. Condition of coral colonies (normal, pale, bleached, overgrown) along line-transects at the water current-exposed and -sheltered site in December 2011 and April 2012 in Gayraca Bay and a water depth of 10 m. Coral condition at the sheltered (A) and the exposed (B) site in December 2011 are displayed in the upper panels and (C) and (D) represent the sheltered and exposed site in April 2012. Abbreviations: *Montastraea cavernosa* (Mcav), *Orbicella faveolata* (Ofav), *Orbicella franksi* (Ofra), *Diploria strigosa* (Dstr), *Diploria labyrinthiformis* (Dlab), *Colpopyllia natans* (Cnat), *Porites astreoides* (Past), *Siderastrea siderea* (Ssid).

During the second coral bleaching monitoring campaign, 6% of the corals bleached at the exposed and 4% at the sheltered site, whereas same coral species were affected at both sites (Fig. 4). Here, no significant difference in relative proportion of overall bleached corals was observed between sites (Table 1). Bleaching was mainly represented by the species *D. strigosa* (3% bleached) at the sheltered site, *M. cavernosa* (15%) at the exposed site and *S. siderea* at both sites (14% at sheltered and 40% at exposed, Fig. 4).

During the first coral bleaching monitoring campaign, significant differences in monitoring time (Table 1) indicated a recovery of corals from bleaching. Whereas 20.8% of the relative coral proportion (pale and bleached; Fig. 2) recovered after upwelling with a rate of 7.0% month^−1^ at the water current-exposed site, a higher pale and bleached coral proportion of 30.8% recovered at the sheltered site resulting in a recovery rate of 6.3% month^−1^ (Fig. 2). An overall better recovery was observed at the exposed site where 97% normal corals were found, whereas only 72% could be identified as normal at the sheltered site and 12% coral cover died here by April 2011.

During the second coral bleaching monitoring campaign, a significant difference between monitoring times (Table 1) indicated that corals recovered from bleaching by the end of upwelling. Here, rates of recovery between sites were similar for the mild bleaching resulting in 3.4% month^−1^ at the exposed and 3.5% month^−1^ at the sheltered site.

The BMI as a measure of coral bleaching response was 0.28 in December 2010 and 0.30 in December 2011 for the exposed site, whereas at the sheltered site, a higher BMI of 0.88 and a similar BMI of 0.28 were registered, respectively. During recovery phases of both years, BMI was lower for the exposed site with 0.03 in March/April 2011 and 0.09 in April 2012 as compared to 0.46 and 0.15, respectively. The CBSI characterizing the specific bleaching susceptibility of the coral community was 8.8 for the exposed and 13.2 for the sheltered site indicating higher susceptibility of coral community to bleaching here.

### Water Column Variables

**Figure 5:**
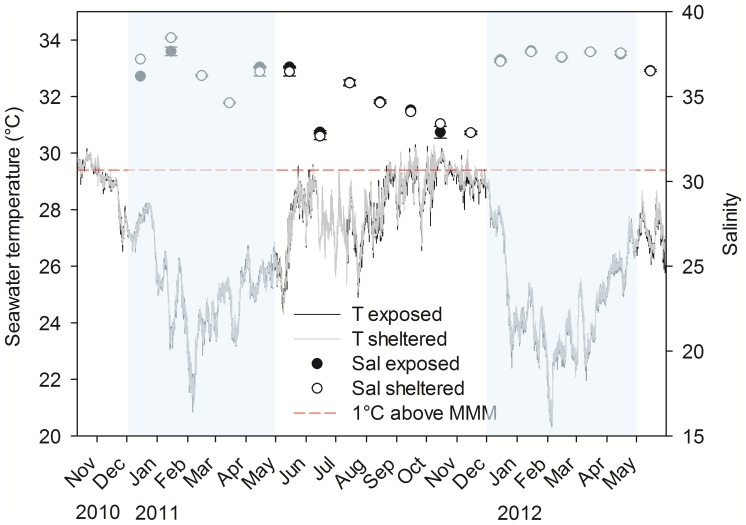
High resolution *in situ* seawater temperature and mean monthly salinity. Seawater temperature with a resolution of 5±0.2°C and salinity as monthly means for the water current-exposed and -sheltered site of Gayraca Bay and a water depth of 10 m are displayed. The coral bleaching threshold of 1°C above maximum monthly mean (MMM) [42] is depicted as a red dashed line. Upwelling months December-April are highlighted in blue.

A seasonality for the TNNP region was deduced from *in situ* seawater temperature of 28.5±1.1°C (mean ± SD) and salinity of 35.3±1.6 during non-upwelling (rainy season) as compared to 25.0±1.7°C and 37.0±0.9, during upwelling (dry season), respectively. Water temperature ranged from 20.3°C (February 2012) to 30.3°C (October 2011) and salinity from 32.5 (October, June) to 38.5 (January; Fig. 5). Water temperature and salinity were significantly different between seasons (Table 2).

**Table 2:** Environmental variables and PERMANOVA results.

	Non-upwelling (mean ± SD)		Upwelling (mean ± SD)		Sites		Seasons	
	exp	she	exp	she	Pseudo-*F*	*p*	Pseudo-*F*	*p*
Temperature (°C)	28.5±1.1	28.4±1.1	25.0±1.7	25.0±1.7	0.000	0.994	79.157	0.001**
Degree-heating days	44	56	0	0	0.153	0.694	10.639	0.001**
Salinity	35.29±1.53	35.29±1.61	37.07±0.94	37.03±0.86	0.002	0.958	24.791	0.001**
Water currents (DF)	13.16±7.66	7.81±4.08	17.03±6.93	9.93±3.94	5.926	0.024*	1.359	0.275
Nitrate (µmol L^−1^)	0.15±0.15	0.31±0.19	1.20±1.03	1.16±1.12	0.022	0.899	19.393	0.001**
Nitrite (µmol L^−1^)	0.06±0.04	0.09±0.06	0.14±0.08	0.14±0.06	0.051	0.814	2.107	0.147
SRP (µmol L^−1^)	0.16±0.14	0.15±0.07	0.14±0.13	0.14±0.12	0.077	0.774	0.005	0.940
Chlorophyll *a* (µg L^−1^)	0.74±0.28	0.87±0.58	2.25±1.59	1.02±0.80	3.314	0.069	8.574	0.004**
Water clarity (m)	11.6±2.1	12.7±2.9	8.5±1.7	11.1±1.9	6.278	0.015*	4.274	0.044*

Environmental variables are displayed as mean ± SD. Levels of significance for differences between sites (exposed vs. sheltered) and seasons (upwelling vs. non-upwelling) are indicated by asterisks with * for significant (p<0.05) and ** for very significant (p<0.01). Abbreviations: exposed site (exp), sheltered site (she), soluble reactive phosphorus (SRP), and diffusion factor index (DF).

A locally-calculated coral bleaching threshold of 29.4°C [42] was used to define degree-heating days as described above. From this calculation, temperature anomalies of 21 degree-heating days at the exposed and 22 at the sheltered site were detected for October-November 2010 (Table 2). Continuous temperature monitoring in high temporal resolution did not reveal any statistical differences between sites. However, in 2011 the temperature anomaly for October-November accounted for 15 degree-heating days at the exposed and 23 at the sheltered site (Table 2). The total temperature anomaly during the rainy season in 2011 was represented by 23 degree-heating days at the exposed and 34 at the sheltered site (Table 3).

**Table 3:** Indices of water temperature.

	Mean temp (°C)		Max temp (°C)		Min temp (°C)		Degree-heating days (>29.4°C) (d)	
Months vs. sites	exp	she	exp	she	exp	she	exp	she
Oct 10	29.63	29.63	30.17	30.04	28.89	29.17	19	20
Nov 10	28.65	28.65	29.84	29.92	26.52	26.77	2	2
*Dec 10*	27.43	27.42	28.25	28.25	25.36	25.38	0	0
*Jan 11*	24.94	24.92	26.52	26.50	22.92	22.82	0	0
*Feb 11*	23.44	23.40	24.75	24.87	20.84	21.08	0	0
*Mar 11*	24.73	24.71	26.26	25.94	23.16	23.23	0	0
*Apr 11*	25.65	25.63	26.84	26.82	24.73	24.77	0	0
May 11	27.10	27.07	29.54	29.27	24.32	24.29	0	0
Jun 11	28.69	27.91	29.59	29.52	26.67	25.62	0	0
Jul 11	27.16	27.43	28.94	29.34	24.90	25.33	0	0
Aug 11	28.27	28.34	29.67	29.74	26.30	26.48	1	3
Sep 11	28.88	28.96	30.29	30.19	26.55	26.72	7	8
Oct 11	29.41	29.49	30.27	30.29	27.43	27.95	14	20
Nov 11	29.03	29.12	30.02	30.12	28.00	28.35	1	3
*Dec 11*	26.44	26.51	28.94	29.07	22.39	22.99	0	0
*Jan 12*	23.88	23.94	25.36	25.43	21.80	21.89	0	0
*Feb 12*	22.69	22.75	24.22	24.48	20.32	20.39	0	0
*Mar 12*	24.07	24.13	25.40	25.55	21.96	22.39	0	0
*Apr 12*	26.13	26.19	27.78	27.78	24.92	24.97	0	0
Non-upwelling (rainy season) 2010	29.04	29.04	30.17	30.04	26.52	26.77	21	22
*Upwelling (dry season) 2010/11*	25.27	25.25	28.25	28.25	20.84	21.08	0	0
Non-upwelling (rainy season) 2011	28.39	28.31	30.29	30.29	24.32	24.29	23	34
*Upwelling (dry season) 2011/12*	24.66	24.72	28.94	29.07	20.32	20.39	0	0

Water temperature indices are displayed with seasonal (upwelling vs. non-upwelling) and spatial (exposed vs. sheltered site) resolution. Upwelling months are indicated by italics. Degree-heating days were calculated from mean daily temperature above local coral bleaching threshold of 29.4°C [42]. Abbreviations: exposed site (exp), sheltered site (she), mean temperature (mean temp), maximum temperature (max temp), and minimal temperature (min temp).

Water currents measured by clod cards were significantly higher at the exposed as compared to the sheltered site according to still water control-normalized clod cards (Table 2).

Nitrate concentrations were significantly increased during upwelling when compared to non-upwelling (Table 2) with 1.20±1.03 µmol L^−1^ at the exposed and 1.16±1.12 µmol L^−1^ at the sheltered site as compared 0.15±0.15 µmol L^−1^ and 0.31±0.19 µmol L^−1^ during non-upwelling. However, no significant differences were found between sites (Table 2).

Chlorophyll *a* concentrations had mean concentrations of 2.25±1.59 µg L^−1^ (mean ± SD) for the exposed and 1.02±0.80 µg L^−1^ for the sheltered site during upwelling and 0.74±0.28 µg L^−1^ and 0.87±0.58 µg L^−1^, respectively. Significant difference was observed between seasons but not between sites (Table 2).

Water clarity was significantly different between the exposed and sheltered site with more turbid water at the exposed site (Table 2). Significant differences between seasons revealed that seawater was more transparent during non-upwelling (Table 2).

## Discussion

### Bleaching Susceptibility and Recovery Patterns of TNNP Corals

In this study, we observed over a time period of two years that hard corals at a water current-sheltered site were more susceptible to coral bleaching than corals at an -exposed site in the upwelling influenced TNNP. Riegl and Piller [17] observed that coral health and recovery from the mass bleaching event in 1998 for the Bahamas was better at sites where a small-scale upwelling was present (70% healthy corals) as compared to nearby located sites governed by down-welling of heated surface water (44% healthy corals). Jiménez et al. [24] showed that corals at locations exposed to oceanic conditions and seasonal upwelling in Costa Rica experienced a reduced warming and consequently less coral bleaching than locations where upwelling was absent. Riegl [25] reported that corals in South Africa were protected against coral bleaching by a seasonal upwelling whenever upwelling and warming coincided. Whereas the mentioned studies correlate a reduced coral bleaching during warming events to seawater temperature decrease, the present study is the first one to show that differences in susceptibility of corals were potentially driven by the degree of exposure to water currents as no temperature differences were detected between the sites in the same upwelling-influenced bay.

The results of our study are further supported by observations of Wall et al. [30] where corals at the Similan Islands in the Andaman Sea (Thailand) at sites sheltered from deep-water intrusions by long amplitude internal waves (LAIW) were more susceptible to bleaching in 2010 (above 50% bleached and recently dead corals) than corals at exposed sites (39 – 45%) characterized by stronger currents and mixing of water masses [43]. Similar to our study, Wall et al. [30] found a difference in community composition of more susceptible coral species at the sheltered sites (*Acropora* spp. and massive *Porites* spp.), whereas the more resistant *Diploastrea* spp. were predominant at the exposed locations. Some of the susceptible coral species represented at both sites (e.g. *Pocillopora* spp. and branching *Porites* spp.) vanished from the sheltered sites and only remained in reduced numbers at the exposed sites [30]. For TNNP, the observed spatial differences in bleaching susceptibility of scleractinian corals during the first coral bleaching monitoring campaign can be partly attributed to the differences in coral community composition as the mainly bleached coral species *O. faveolata* and *O. franksi* only occurred at the sheltered site. Bleaching at the exposed site was represented by *M. cavernosa* and *D. labyrinthiformis*. Multivariate regression analyses of coral communities at sheltered eastern and exposed western sites in four consecutively located bays in TNNP (including Gayraca Bay) revealed that the specific community composition between sites depended mainly on exposure characterized by strong northeast winds, waves and resulting water currents among the tested factors (exposure, bay, season during monitoring, water temperature, temperature variance, and salinity; Eidens et al. unpublished data). Indicator species analyses furthermore implied that the reef-building but bleaching-susceptible *Orbicella* spp. almost exclusively occurred at the sheltered sites within all bays, while the exposed sites were dominated by encrusting forms of *Diploria* spp. and *S. siderea* (Eidens et al. unpublished data). In Gayraca Bay, *Orbicella* spp. accounted for 41% of the overall coral community at the sheltered site. This characteristic coral community pattern was also observed by Werding and Sánchez [44] who suggested a difference in wave exposure gradient between two opposing sites of one TNNP bay to be the causal factor for the specific coral community composition between sites. The results of the present study suggest that differences in water currents exposure shape the specific coral community distribution and have thereby an indirect effect on the different coral bleaching responses between the exposed and sheltered site.

The massive *O. faveolata* and *O. franksi* that were severely affected by coral bleaching in 2010 are particularly bleaching-susceptible species in the Caribbean and TNNP [31], [33]. However, bleaching of the latter species was largely absent during the second coral bleaching monitoring campaign of the present study. Here, the affected corals were *D. strigosa* and *S. siderea* for the sheltered and *M. cavernosa* and *S. siderea* at the exposed site, but differences in bleaching of overall coral cover between sites were insignificant.

Corals of the genus Orbicella (formerly known as Montastraea [34]) play a crucial role in reef accretion, thereby providing a framework to a vast variety of organisms in the wider Caribbean [45]. The complete absence of bleaching in 2011 for Orbicella that survived bleaching in 2010 may be a result of rapid acclimatization e.g. via a shift to a more resistant symbiotic community of the coral host [46]–[50], through the expression of heat shock proteins which repair denatured cellular components [51], antioxidant enzymes inactivating harmful oxygen radicals [52]–[54], photoprotective fluorescent proteins that reflect and dissipate excess light [55], ultraviolet radiation-absorbing mycosporine-like amino acids [56], and increase of host tissue thickness [57] that play an important role in the regulation of coral bleaching response.

Corals recovered by March/April, 5 – 6 months after the moderate coral bleaching end of 2010 and mild bleaching event in 2011. During time of recovery, seawater temperature decreased below 21°C during both years of study. Coral recovery in TNNP was faster than the recovery time of 6 – 8 months characterized as a good recovery after the 1998 severe bleaching event of the Great Barrier Reef [58]. Longest recovery periods after severe coral bleaching events can take up to 13 years [59]. In contrast to the study of Wall et al. [30], where no differences in coral recovery were observed between LAIW-exposed and –sheltered sites of the Similan Islands/Andaman Sea, we observed a faster and more efficient coral recovery at the water current-exposed site as compared to the -sheltered counterpart where a coral mortality of 12% occurred. However, data on coral bleaching and recovery cannot be compared to recent previous coral bleaching events in the TNNP so far, as the extent of bleaching during the last decade was negligible [6], [60], [61].

### Potential Reasons for the Observed Bleaching Patterns

Our findings suggest that the overall coral community bleaching was mitigated at a water current exposed as compared to a sheltered site of the same upwelling influenced bay in TNNP. The following possible explanatory factors were examined in detail: (1) decrease of seawater temperature after or during time of highest heat stress; (2) reduced temperature anomalies above bleaching threshold; (3) increased water current exposure through stronger winds during upwelling; and (4) increased turbidity that may reduce light/UV stress.

Several studies have discussed the coral bleaching mitigation effect of upwelling-induced decrease of water temperature during time of highest heat stress [15], [17], [18]. However, in our study the differences in absolute water temperatures between the exposed and sheltered site of Gayraca Bay were insignificant and the calculated temperature anomalies between sites were comparable. Consequently, our data indicate that water temperature decreases were not mainly responsible for the observed coral bleaching and recovery dynamics between the investigated sites.

The key reason for the observed coral bleaching and recovery patterns is likely the difference in exposure to water currents at the investigated locations. This is supported by the study of Nakamura and van Woesik [62] in which bleaching of staghorn corals during the 1998 bleaching event was more pronounced at water current exposure-sheltered compared to -exposed locations close to Ryukyu Island (Japan). The observed findings in the present study are also in accordance with Nakamura et al. [63], demonstrating that a recovery was facilitated for the artificially bleached hard coral *Stylophora pistillata* through high water current exposure. Water currents can generally influence coral physiology and performance [16], [62], [63]. Possible reasons are that water current-induced mass exchange and transport processes are orders of magnitude faster than the exchange via molecular diffusion in still water [64] leading to increase in particulate organic matter release [65], enhanced photosynthesis [66] and ammonium uptake rates in corals [67]. Water currents may mitigate coral bleaching by rapid removal of toxic reactive oxygen species (ROS), nitric oxides, and their permeable derivatives [53], [68] from the host's and symbionts' cells [62]. This may explain the reduced overall coral community bleaching and increased recovery detected at the water current-exposed site in Gayraca Bay. Some studies suggested that the difference in water currents and wave-exposition may mediate the reef community structure [44], [69] which in turn defines the community susceptibility to coral bleaching. This may explain the higher community bleaching susceptibility index observed at the water current-sheltered as compared to the -exposed site. In accordance with Werding and Sánchez [44], the present study suggests that water current exposure influences the specific coral community composition between the exposed and sheltered sites in TNNP and thereby has also an indirect effect on differences in coral bleaching response between sites.

A further explanation for the observed findings may be the increased turbidity at the water current-exposed site, likely due to resuspension of fine sediments. Regions with increased turbidity [70], [71] and high cloud cover [72] exhibit low bleaching susceptibility, which consequently supports our observations of more resilient corals at the water current-exposed site with higher turbidity as compared to the -sheltered site.

The described extrinsic environmental factors and the intrinsic, species- or colony-specific factors can be emphasized as mechanisms to promote coral resistance and resilience [16]. We consider the differences in water currents as major local control for the coral ecosystem in TNNP and suggest that they potentially determine the characteristic species distribution between the exposed and the sheltered sites of the bays, reported by various studies [10], [27], [44].

### The Potential Role of Seasonal Upwelling in Coral Bleaching Mitigation

Before 2010, significant coral bleaching events were absent for decades in TNNP [6], [7]. During the same time in 2010 that we observed a moderate coral bleaching for the upwelling influenced TNNP, massive coral bleaching events were detected in Puerto Cabello [73] and Los Roques National Park in Venezuela [74] which experience no seasonal upwelling [12]. Here, up to 63% bleached corals were reported for Puerto Cabello [73] and 72% bleached and pale coral colonies for Los Roques National Park [74]. Bleaching extent in these non-upwelling regions was more than twice as much as at the water current-sheltered and 7 – 9 times more than at the -exposed site in our study. These findings, along with the absence of coral bleaching in the past, suggest that seasonal upwelling potentially plays a role in coral bleaching mitigation in TNNP. Coral bleaching in Puerto Cabello was correlated to increased seawater temperature to 31°C due to El Niño forcing and a subsequent La Niña phase of the El Niño Southern Oscillation which drastically decreased salinity in the reef seawater by strong rainfalls [73], also present in the TNNP region. In Los Roques National Park, high coral bleaching extent which accounted for the first mass bleaching event in 20 years for the region, was explained by unusually high thermal anomalies of more than 16 degree-heating weeks (DHW) [74]. The authors [74] suggested that Los Roques was massively impacted in 2010 because the region was lacking significant coral bleaching events in the past. So far, there is some evidence that susceptibility of corals to bleaching depends on the thermal history [75]–[77] of coral reefs and consequently on former bleaching events [78]. Coral bleaching extent and/or frequency of bleaching events may be higher for regions without coral bleaching history when seawater temperatures rise above coral bleaching thresholds [78]. This partly explains the moderate coral bleaching in Gayraca Bay observed in the present study.

During the Caribbean Crisis in 2005, Rodríguez-Ramírez et al. [79] attributed the minimal coral bleaching occurrence of 1 – 5% coral cover in TNNP to seasonal upwelling. Later, Chollett et al. [18] suggested the TNNP region as potential refuge for corals against future coral bleaching events as typically heating and upwelling-induced cooling of seawater are in synchrony. However, during the El Niño impacted year 2010, heating started anomalously earlier than expected with the consequence that coral bleaching threshold of 4 DHW was already exceeded during September as compared to previous years or the second coral bleaching monitoring where accumulation of thermic stress for scleractinian corals started in November-December. In addition, during 2010, a record heat accumulation of 12 DHW was reported during November as compared to previous years (maximum 7 DHW; http://coralreefwatch.noaa.gov/). A typical pattern of upwelling-influenced coral bleaching mitigation may have been present during the second coral bleaching monitoring campaign where a maximum experienced heat stress of 4 DHW coincided with onset of seasonal upwelling. Thereby, the present study also supports the hypothesis formulated by Glynn [15] that upwelling areas could act as possible refuge habitats in which corals are protected from temperature-induced coral bleaching.

### Ecological Implications

In our study, we identified direct and indirect water current-induced coral bleaching mitigation for an exemplary bay in the upwelling influenced TNNP. This study thereby indicates the existence of local resilience patterns against coral bleaching for reefs in TNNP. Compared to other nearby non-upwelling locations in the Caribbean such as Islas del Rosario, Islas de San Bernardo (Colombia; [6]) or Puerto Cabello and Los Roques National Park (Venezuela [73], [74]), where coral bleaching was more severe, the bays of the TNNP may act as refugia for corals from bleaching in times of ocean warming due to global climate change.
